# Supplementary material for: Causal Relationship between Aging and Anorexia Nervosa: A White-Matter-Microstructure-Mediated Mendelian Randomization Analysis
Source: Biomedicines. 2024 Aug 16;12(8):1874. doi: 10.3390/biomedicines12081874 (PMC11351342; doi:10.3390/biomedicines12081874)
Supplement: Supplementary file 1 [file biomedicines-12-01874-s001.zip › Supplementary Figure/Figure S4/Singe SNP plot of MR analysis for TL to ubm-b-2087.pdf]

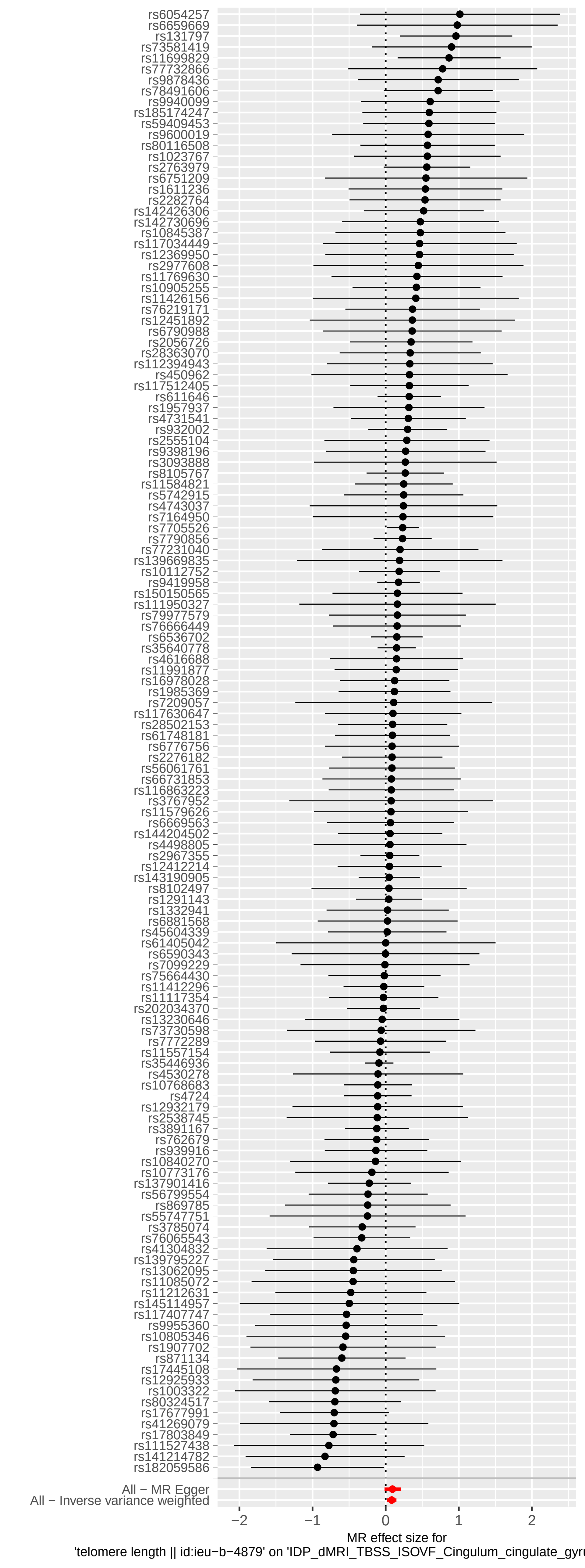

rs6054257
rs6659669
rs131797
rs73581419
rs11699829
rs77732866
rs9878436
rs78491606
rs9940099
rs185174247
rs59409453
rs9600019
rs80116508
rs1023767
rs2763979
rs6751209
rs1611236
rs2282764
rs142426306
rs142730696
rs10845387
rs117034449
rs12369950
rs2977608
rs11769630
rs10905255
rs11426156
rs76219171
rs12451892
rs6790988
rs2056726
rs28363070
rs112394943
rs450962
rs117512405
rs611646
rs1957937
rs4731541
rs932002
rs2555104
rs9398196
rs3093888
rs8105767
rs11584821
rs5742915
rs4743037
rs7164950
rs7705526
rs7790856
rs77231040
rs139669835
rs10112752
rs9419958
rs150150565
rs111950327
rs79977579
rs76666449
rs6536702
rs35640778
rs4616688
rs11991877
rs16978028
rs1985369
rs7209057
rs117630647
rs28502153
rs61748181
rs6776756
rs2276182
rs56061761
rs66731853
rs116863223
rs3767952
rs11579626
rs6669563
rs144204502
rs4498805
rs2967355
rs12412214
rs143190905
rs8102497
rs1291143
rs1332941
rs6881568
rs45604339
rs61405042
rs6590343
rs7099229
rs75664430
rs11412296
rs11117354
rs202034370
rs13230646
rs73730598
rs7772289
rs11557154
rs35446936
rs4530278
rs10768683
rs4724
rs12932179
rs2538745
rs3891167
rs762679
rs939916
rs10840270
rs10773176
rs137901416
rs56799554
rs869785
rs55747751
rs3785074
rs76065543
rs41304832
rs139795227
rs13062095
rs11085072
rs11212631
rs145114957
rs117407747
rs9955360
rs10805346
rs1907702
rs871134
rs17445108
rs12925933
rs1003322
rs80324517
rs17677991
rs41269079
rs17803849
rs111527438
rs141214782
rs182059586
All − MR Egger
All − Inverse variance weighted
−2
−1
0
1
2
MR effect size for
'telomere length || id:ieu−b−4879' on 'IDP_dMRI_TBSS_ISOVF_Cingulum_cingulate_gyru
